# Long-term memory representations for audio-visual scenes

**DOI:** 10.3758/s13421-022-01355-6

**Published:** 2022-09-13

**Authors:** Hauke S. Meyerhoff, Oliver Jaggy, Frank Papenmeier, Markus Huff

**Affiliations:** 1 University of Erfurt, Nordhäuser Str. 63, 99089 Erfurt, Germany; 2 Leibniz-Institut für Wissensmedien, Tübingen, Germany; 3 Department of Psychology, University of Tübingen, Tübingen, Germany

**Keywords:** Long-term memory, Audio-visual integration, Study-test congruency, Audio-visual advantage, Naturalistic scenes

## Abstract

In this study, we investigated the nature of long-term memory representations for naturalistic audio-visual scenes. Whereas previous research has shown that audio-visual scenes are recognized more accurately than their unimodal counterparts, it remains unclear whether this benefit stems from audio-visually integrated long-term memory representations or a summation of independent retrieval cues. We tested two predictions for audio-visually integrated memory representations. First, we used a modeling approach to test whether recognition performance for audio-visual scenes is more accurate than would be expected from independent retrieval cues. This analysis shows that audio-visual integration is not necessary to explain the benefit of audio-visual scenes relative to purely auditory or purely visual scenes. Second, we report a series of experiments investigating the occurrence of study-test congruency effects for unimodal and audio-visual scenes. Most importantly, visually encoded information was immune to additional auditory information presented during testing, whereas auditory encoded information was susceptible to additional visual information presented during testing. This renders a true integration of visual and auditory information in long-term memory representations unlikely. In sum, our results instead provide evidence for visual dominance in long-term memory. Whereas associative auditory information is capable of enhancing memory performance, the long-term memory representations appear to be primarily visual.

## Introduction

At any waking moment, an endless stream of multimodal sensory information shapes our mental representation of the outside world. Over the past two decades, more and more research has appreciated the multimodal nature of human perception, as well as crossmodal interactions between different sensory streams. Whereas visual information processing was originally supposed to be impenetrable by non-visual information such as audition (e.g., Rock & Victor, 1964; Warren et al., 1981), more recent research has revealed strong and persistent interactions between auditory and visual information during perception (for reviews, see Ernst & Bülthoff, 2004; Koelewijn et al., 2010; Spence, 2011) as well as short memory durations (for reviews, see Matusz et al., 2017; Shams et al., 2011). However, research that has investigated how the interplay of multiple modalities forms subsequent long-term memory representations in humans is rare (see Gibson & Maunsell, 1997, for evidence from non-human primates). In the following, we report a modeling approach as well as behavioral experiments that investigate the nature of human long-term memory representations for brief naturalistic scenes. More specifically, we ask whether these memory representations consist of integrated auditory and visual information or whether auditory and visual information contribute to long-term memory independently of each other. As evidence in favor of audio-visual integration, we would consider long-term memory performance that cannot be explained by independent contributions of auditory and visual information.

### Audio-visual integration during perception

There is a continuous interplay between perceptual processes and long-term memory. Whereas perception is obviously necessary to acquire new long-term memory representations, previously established long-term memory representations influence ongoing perceptual and attentional processes (e.g., Biederman et al., 1982; Võ & Wolfe, 2013).

Regarding perceptual processes, auditory information is capable of altering the quantity as well as the quality of a visual percept. For instance, when two brief sounds coincide with one visual flash, observers tend to perceive two visual flashes (Shams et al., 2000), and when a brief sound coincides with the moment of overlap between two moving discs, observers tend to misperceive the spatial relations between the moving discs (Meyerhoff & Scholl, 2018), resulting in the impression of two discs bouncing off rather than streaming past each other (Sekuler et al., 1997). Importantly, this crossmodal influence is not unidirectional, but both sensory streams are integrated into one joint percept (e.g., Alais & Burr, 2004; McGurk & MacDonald, 1976). In fact, there is neuroanatomical (Falchier et al., 2002) as well as electrophysiological evidence (e.g., Giard & Peronnet, 1999; van der Burg et al., 2011) that the integration of auditory and visual signals starts at the earliest stages within the cortex. Crucially, however, the effectiveness of this integration process strongly depends on the synchrony of both signals with a tolerance of only ± 200 ms (Lewald et al., 2001; Meyerhoff & Suzuki, 2018; Powers et al., 2009; Stevenson et al., 2012; van Wassenhove et al., 2007).

It seems obvious that perceptual illusions also affect subsequent memory representations; however, audio-visual interactions might also influence subsequent memory representations less obviously by increasing the efficiency of sensory processing in one of the modalities. Indeed, there is a substantial body of research demonstrating such early interactions between semantically meaningful auditory information and visual perception (see also Taylor et al., 2009, for neuropsychological evidence). With auditory information preceding the visual stimuli by a few hundred milliseconds, congruent naturalistic sounds (Chen & Spence, 2011a, b) as well as spoken words (Chen & Spence, 2018; Edmiston & Lupyan, 2015; Lupyan & Thompson-Schill, 2012; Lupyan & Ward, 2013) facilitate the detection of the corresponding visual objects. However, even when auditory and visual information is presented in temporal alignment, sounds still enhance visual processing relative to unimodal or semantically mismatching audio-visual presentations. For instance, semantically matching sounds accelerate the fixation (Iordanescu et al., 2010) and detection (Iordanescu et al., 2008) of visual objects, as well as facilitate their identification (Amedi et al., 2005; Chen & Spence, 2011b; Mädebach et al., 2017). Remarkably, this holds true even when conscious processing of the visual scene is prevented (Tan & Yeh, 2015).

Furthermore, audio-visually synchronous events attract attention (Meyerhoff et al., 2022; Santangelo & Spence, 2007; van der Burg et al., 2008), thus further improving their processing. This enhanced processing also impacts subsequent processes. For instance, perceptual learning (i.e., performance improvements with practice in basic perceptual tasks) is more pronounced following multimodal presentations than unimodal presentations alone (Kim et al., 2008; Seitz et al., 2006). Likewise, it therefore seems plausible that audio-visually integrated information also shapes short-lived memory representations as well as long-term memory representations.

### Audio-visual impact on working memory

Similar to perceptual processes, the ability to store information in working memory appears to benefit from multimodal stimulus presentations. For instance, Frick (1984) demonstrated an increased capacity limitation for digits when the information was distributed between the visual and auditory modality. Importantly, observers also recalled more information from working memory when auditory and visual information had been presented simultaneously rather than unimodally in isolation (Delogu et al., 2009; for similar results, see also Goolkasian & Foos, 2002; Lewandowski & Kobus, 1993). However, the critical question, namely, whether the benefits of multimodal information in working memory stem from audio-visual integration (Saults & Cowan, 2007) or independent storages for auditory and visual information (Baddeley & Logie, 1999; Fougnie & Marois, 2011; for a review, see Quack et al., 2015), has not yet been resolved. Indeed, there is also experimental evidence that limits the general validity of the advantage of audio-visual stimuli in memory. Depending on the moment of retrieval, it has also been shown that either visual (Ngo et al., 2010) or auditory information alone (Ngo et al., 2010) might dominate the remaining modality.

In a related line of research, Murray and colleagues (for a review, see Matusz et al., 2017) have been studying the impact of audio-visual presentations on unimodal retrieval using the continuous recognition paradigm. In this paradigm, the participants attended to a stream of briefly presented stimuli, indicating whether the currently presented stimulus had been presented among the previously attended stimuli. Whereas the repeated items were unimodal, the initial presentation of the stimuli could have been either multimodal or unimodal as well. Semantically congruent audio-visual information during the initial presentation had a beneficial effect on the recognition of visual (e.g., Lehmann & Murray, 2005; Thelen et al., 2015) as well as auditory information (e.g., Matusz et al., 2015; Thelen et al., 2015). Using electrophysiology (Murray et al., 2004) as well as neuroimaging techniques (Murray et al., 2005), it was demonstrated that visual object identification was more active during unimodal recall for stimuli with rather than without a multimodal history (see also von Kriegstein & Giraud, 2006). As there were no general differences in performance for unimodal and audio-visual stimuli at the initial presentation (e.g., Matusz et al., 2015), the authors argued that the improved recognition performance does not stem from general enhancements of attentional or encoding-related processes. This is further supported by the observation that the presence of meaningless tones during the initial presentation could have detrimental effects on subsequent unimodal recognition (e.g., Lehmann & Murray, 2005; Thelen et al., 2012). Please note, however, that a recent re-investigation has revealed little evidence for the generality of a beneficial impact of audio-visual encoding on visual recognition performance (Pecher & Zeelenberg, 2022).

### Auditory and visual information in long-term memory

With regard to long-term memory (i.e., long retention intervals with clearly separated encoding and recall/recognition), auditory and visual material has been studied in isolation rather than in combined presentation formats. This research has revealed a tremendous capacity for visual memory representations. In fact, no capacity limitation has been reported yet. For instance, Standing (1973) observed a linearly increasing number of successfully recognized images up to 10,000 pictures, which was the maximum of presented items in his study. This tremendous capacity is not restricted to pictures but also arises with real-world objects at a remarkable level of detail (Brady et al., 2008; Brady et al., 2013) as well as with the location of objects within entire scenes (Hollingworth, 2004, 2005; Konkle et al., 2010). Further, visual long-term memory representations not only consist of static object information, but also contain dynamic information such as changes over time in a dynamic scene. Using brief excerpts from movies, Matthews et al. (2007); see also Goldstein et al., 1982) demonstrated that observers are more accurate in discriminating previously studied dynamic clips from novel clips than their static counterparts (or a series of static snapshots). This dynamic superiority effect still appeared with retention intervals of four weeks. Importantly, however, recognition performance was best when the clips were tested in the same dynamic state as they had been presented during the study session (Buratto et al., 2009; Matthews et al., 2010). In other words, dynamic scenes were recognized more accurately when they were tested dynamically rather than statically, and their static counterparts were recognized more accurately when they were tested statically rather than dynamically (i.e., study-test congruency). This finding indicates that the dynamic information is in fact part of the memory representation as presenting this information additionally during testing hurts the recognition performance for statically studied scenes.

Compared to visual memory, memory for auditory stimuli appears to be inferior (Cohen et al., 2009; see also Kassim et al., 2018); however, recent work from our group has demonstrated that information from both modalities interacts during the formation of long-term memory representations (Meyerhoff & Huff, 2016). In this study, the participants studied brief auditory, visual, or audio-visual tracks from movies. Recognition performance following retention intervals of 1 day or 1 week was in fact more accurate for audio-visual tracks than for their unimodal counterparts. However, this does not necessarily indicate audio-visual integration as audio-visual tracks also contain more retrieval cues that could improve memory performance independently of each other. One finding supporting the hypothesis of audio-visually integrated long-term memory representations was that semantically matching stimuli were recognized more accurately than mismatching stimuli (i.e., visual and auditory information from different movie clips). Nevertheless, even these semantically mismatching audio-visual tracks were recognized more accurately than purely visual tracks (i.e., unimodal). This indicates that visual and auditory information are both also capable of improving memory performance independently of each other. Finally, the role of audio-visual synchrony deviated remarkably from what would be predicted from the studies on audio-visual integration during perception (see above). Whereas audio-visual synchrony is a key variable during perception, it was strikingly irrelevant in this study. Even when matching auditory and visual tracks were presented sequentially, recognition performance remained at the level of tracks presented in audio-visual synchrony. Only when multiple tracks were presented in between the matching auditory and visual tracks, did recognition performance finally decline during audio-visual testing relative to the tracks that had been studied in audio-visual synchrony. In sum, this study demonstrates that auditory information can improve memory for visual scenes (i.e., the effect of semantic congruency); however, it remains inconclusive whether this advantage actually stems from an integration of auditory and visual information in long-term memory representations.

### Interplay of auditory and visual information in long-term memory representations

**Fig. 1:**
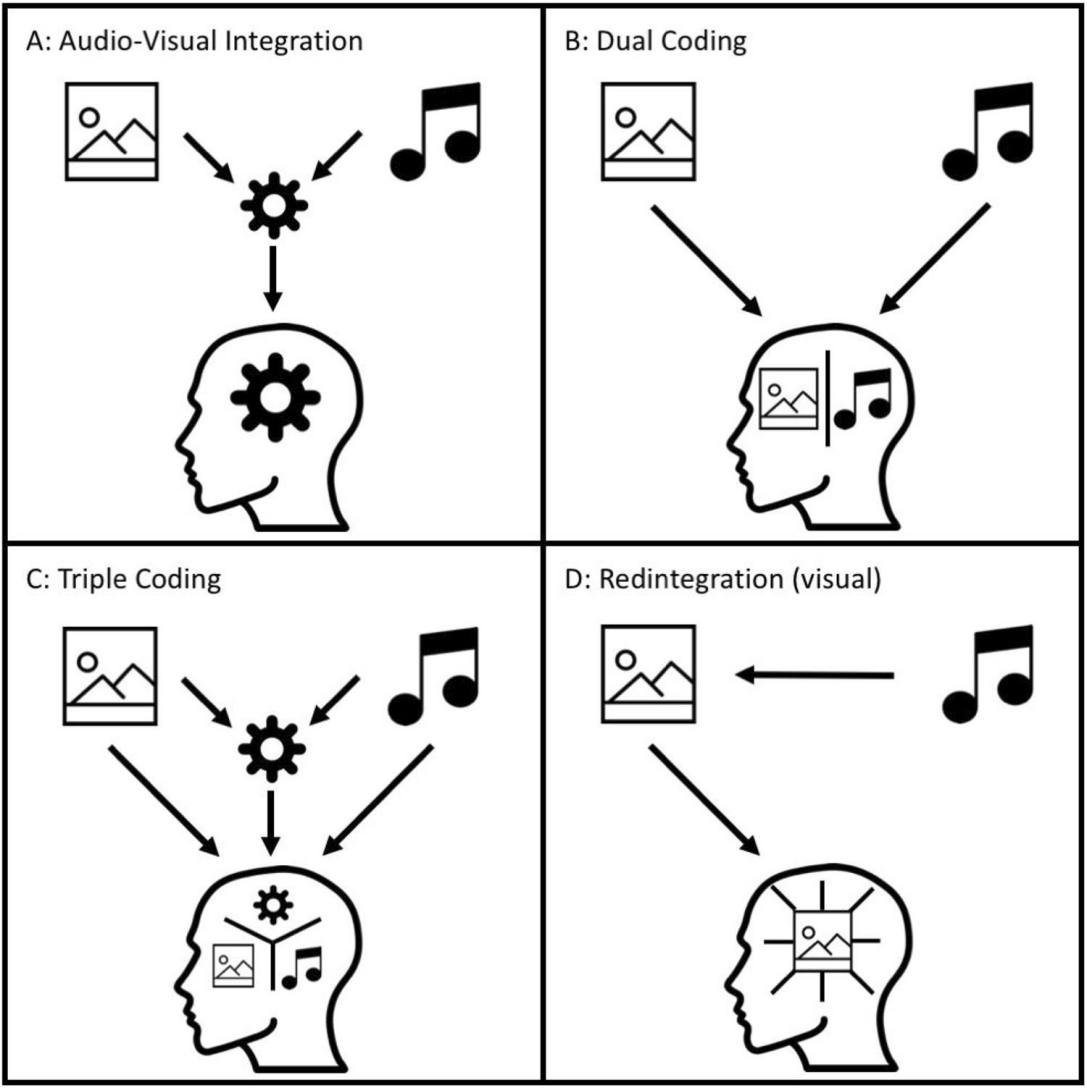
Depiction of the central theoretical accounts addressing the interplay of auditory and visual information within memory representations. **A:** Audio-visual integration assumes that both modalities are merged into a unitary, amodal representation. **B:** Dual coding assumes that both modalities are encoded and stored separately. **C:** Triple coding assumes memory representations consist of auditory, visual, and integrated, amodal scene components. **D:** Redintegration assumes that audio-visual interactions enhance memory representations, which remain accessible during unimodal testing (depicted for visual memory). Icons originally designed by Smashicons from Flaticon

Across different research traditions and paradigms addressing the interplay of distinct sources of information in memory representations, several theories have been suggested in order to describe the structure of memory representations. Whereas many of the older studies have asked how verbal and pictorial information interact in memory, more recent work has more directly investigated the interplay of auditory and visual information (for a schematic overview, see Fig. 1).

Regarding the interplay of verbal and pictorial information, some accounts have proposed that memory representations are abstract and amodal in nature (i.e., modality-independent; Anderson, 1978, 1979; Kieras, 1978). However, Paivio and colleagues (Paivio & Csapo, 1973; Thompson & Paivio, 1994) observed that memory performance for audio-visual material in a recall task did not differ from what would be expected based on independent memory representations for the auditory and visual information. According to their interpretation and the corresponding *dual-coding theory* (i.e., modality-specific), the assumption of audio-visual integration in any form is therefore not necessary to explain memory performance. Since the empirical evidence remained conflicting with regard to modality-specific and modality-independent explanations across different tasks and material, there have also been suggestions for *triple code models* that include the modality-specific information as well as integrated information from the different representation formats (e.g., Glucksberg, 1984; Snodgrass, 1984; see also Dehaene, 1992; Dehaene et al., 1999).

Regarding the more direct interplay of auditory and visual information, the theory of redintegration (see Baddeley, 2007) has been proposed to explain the benefit of combined auditory and visual information relative to unimodal information. According to this theory, encoding congruent audio-visual stimuli results in more sophisticated memory representations than encoding auditory or visual stimuli in isolation. Importantly, this memory representation can be (fully) activated by unimodal retrieval cues (e.g., Heikkilä et al., 2015). Empirical support for this theory derives/comes from the studies by Murray and colleagues, who demonstrated more accurate recognition performance for visually tested stimuli that had been studied audio-visually rather than purely visually (e.g., Matusz et al., 2017; Thelen et al., 2015; but see Pecher & Zeelenberg, 2022). Interestingly, there is neuroimaging evidence suggesting that auditory areas of the brain are more active during visual retrieval of stimuli that were studied audio-visually than during visual retrieval of stimuli that were studied visually (Nyberg et al., 2000; Wheeler et al., 2000). However, these studies were not designed to detect behavioral differences in memory accuracy. Therefore, whether the increase in neural activation also elicits more accurate memory accuracy – to our knowledge – is still an open question.

### Rationale of the current project

In this project, we aimed to investigate whether there is evidence for the integration of auditory and visual information during the formation of long-term memory representations. Approaching an answer to this question potentially could help to distinguish between the four theories presented in Fig. 1. All the theories with the exception of the dual-coding theory rely on the assumption that auditory and visual information are integrated (at least to some extend). As integration is a more complex mechanism than independent storages for auditory and visual information, empirical support for such integration is necessary to justify the assumption of integration processes. Surprisingly, however, this assumption has rarely been studied in the context of long-term memory (see Meyerhoff & Huff, 2016). We therefore address this lack of evidence with long-term memory experiments in which we tested memory performance for brief excerpts from movies in order to achieve a good balance between experimental control and ecological validity.

In particular, we were interested in two predictions that follow from the assumption of audio-visually integrated long-term memory representations. The first prediction addresses the recognition performance for audio-visual scenes relative to their unimodal counterparts. In particular, we ask whether the superior memory performance for audio-visual scenes relative to purely visual or purely auditory scenes (Meyerhoff & Huff, 2016) is large enough to rule out explanations based on the simple summation of retrieval cues. While multimodal integration, triple coding, and redintegration implicitly assume a non-additive summation, the presence of such a substantial increase potentially could rule out the dual-coding account. In order to address this prediction, we have reanalyzed the results of the first experiment by Meyerhoff and Huff (2016; retention intervals of 1 day or 1 week) using a Bayesian framework testing whether recognition performance for audio-visual scenes exceeds the performance expected from the recognition performance for auditory or visual tracks alone. To anticipate our results, the observed memory advantage of audio-visual scenes will not be large enough to rule out dual coding.

The second prediction of audio-visually integrated memory representations (e.g., amodal; Anderson, 1978, 1979; Kieras, 1978) addresses the effect of study-test congruency for unimodal versus audio-visual scenes. Of particular interest is the recognition performance for unimodal visual scenes. Whereas all theoretical accounts of long-term memory predict that scenes studied and tested audio-visually elicit the most accurate memory performance (due to more retrieval cues in this condition), different predictions arise for the recognition of visual tracks that had been studied visually or audio-visually. First, multimodal integration predicts that deviations between study and test format cause less accurate recognition performance. For instance, and audio-visual representation in long-term memory would mismatch with a purely visual representation during the test session. Second, dual coding would predict that the unimodal information remains available after encoding audio-visual scenes because both modalities are stored independently. With regard to recognizing a visual or an auditory track, it should therefore be irrelevant if this track was studied unimodally or audio-visually. Third, redintegration would predict more accurate recognition performance for unimodal scenes that have been studied audio-visually as the additional information during encoding should result in a more sophisticated memory representation. We tested these predictions with a series of five experiments in which we manipulated the congruency of the presented modalities between study and test (this method has been successfully used to demonstrate that dynamic information as well as the viewing conditions during encoding are preserved in memory; see Buratto et al., 2009; Reingold, 2002; for a review, see Kent & Lamberts, 2008). To anticipate our results, they will mostly be consistent with the predictions from the dual-coding approach.

## Modelling of recognition performance for audio-visual tracks

**Fig. 2:**
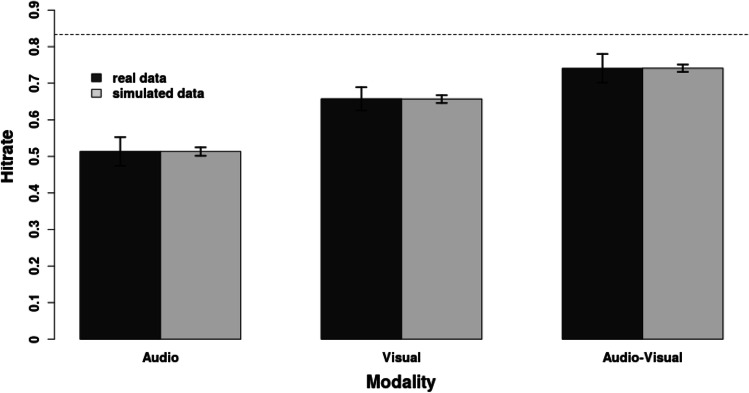
Depiction of the recognition advantage of audio-visual scenes relative to their unimodal counterparts reported in Meyerhoff and Huff (2016). The dark gray bars display the data from the original experiment (the error bars indicate within-subject confidence intervals). The light gray bars display the simulation data of the present study (the error bars indicate highest density intervals). The dashed line represents the criterion of audio-visual integration. Audio-visual memory performance above this line cannot be explained by independent memory traces for auditory and visual information

In the first part of this project, we test whether the recognition performance for audio-visual clips exceeds the threshold at which it cannot be explained with the assumption of independent memory representations for both modalities (i.e., with the availability of multiple retrieval cues). Previous research from our own lab (Meyerhoff & Huff, 2016, Experiment 1; data depicted in the dark bars of Fig. 2) has shown that audio-visual tracks elicit more accurate recognition performance than the unimodal counterparts of these clips. In this experiment, 48 participants performed an old/new recognition task with 900 brief excerpts from Hollywood movies (these excerpts are selected identically to those in the later experiments of this project; see the *Methods* of Experiment 1a for full details). One half of the clips were presented in a study session, whereas the full set was presented in the testing session after a retention interval of 1 day or 1 week. One-third of all stimuli were presented audio-visually (auditory and visual track were semantically congruent, i.e., from the same scene), one-third visually, and the remaining third auditorily during both study and test (i.e., maintaining study-test congruency). The stimuli were counterbalanced across participants so that each clip appeared in each modality equally often. The results showed the most accurate recognition performance for the audio-visual clips, followed by the visual tracks that were recognized more accurately than the auditory clips. Within this reanalysis, we tested whether the benefit of audio-visual clips relative to visual and auditory tracks is large enough to rule out explanations based on independent retrieval cues (Thompson & Paivio, 1994; see also Stevenson et al., 2014). To anticipate our results, the reanalysis will show that memory performance for audio-visual clips is substantially smaller than predicted by the criterion of audio-visual integration.

### Methods

In order to test whether the benefit of audio-visual clips is large enough to rule out independent retrieval cues, we tested whether the hit rate for the audio-visual clips met the integration criterion proposed by Thompson and Paivio (Thompson & Paivio, 1994; see also Stevenson et al., 2014).^1^ According to this criterion, performance gains stem from audio-visual integration if the probability to detect an audio-visual stimulus is larger than the complement probability of neither detecting the unimodal visual nor the unimodal auditory stimulus. Transferred to our current analysis of old/new recognition performance, this criterion states that dual coding could be ruled out if the probability of recognizing audio-visual clips is larger than recognizing the auditory and/or visual track of that clip independently of each other (this threshold is indicated by the dashed line in Fig. 2). In other words, a miss would occur only if a participant fails to recognize the visual as well as the auditory track of a clip. These considerations translate to Equation 1:

1 $$\hat{p}(av)=p(a)+p(v)-p(a)\ p(v)$$

Within this equation, *p̂(av)* refers to the estimated recognition performance if both modalities contribute to the hit rate independently of each other. Please note that performance up to this criterion would not need any additional assumption about audio-visual integration in order to explain the observed performance. Therefore, a clear demonstration of audio-visual integration would require recognition performance of audio-visual clips to exceed that criterion (Equation 2).

2 $$p(av)>\hat{p}(av)$$

We used a Bayesian modeling approach (Gelman et al., 2013; Kruschke, 2014; the modeled data is depicted in the light gray bars of Fig. 2) to estimate the criterion for audio-visual integration (1) as well as to test how the observed hit rates relate to that criterion (2). Following on from Equation 1, the criterion of audio-visual integration within this Bayesian framework could be expressed as in Equation 3.

3 $${\theta}_{crt}={\theta}_a+{\theta}_v-{\theta}_a\ {\theta}_v$$

This approach allows us to simultaneously estimate the parameters for the auditory hit rate, the visual hit rate, the audio-visual hit rate, as well as the criterion for audio-visual integration given our data (y) from the preceding memory experiment (Equation 4)

4 $${\theta}_a,{\theta}_v,{\theta}_{av},{\theta}_{crt} \mid y$$

The relevant trials for this analysis were test trials presenting previously studied items (i.e., hits and misses). For these trials, the observed performance in the old/new recognition task is either a hit or a miss (i.e., a binomial variable). Therefore, the likelihood of our data could be expressed as in Equation 5, in which *θ*_*m*_ is the probability of success in the modality *m ∈ {a, v, av}*, *n*_*m*_ is the number of trials and *y*_*m*_ is the number of hits.

5 $$p\left({y}_m\ |\ {\theta}_m\right)=\left(\ \begin{array}{c}{n}_m\\ {}{y}_m\end{array}\ \right)\ {\theta}_m^{y_m}{\left(1-{\theta}_m\right)}^{n_m-{y}_m}$$

In order to allow for an interpretation of the modeled *θ*_*crt*_, we added an indicator variable signaling whether the hit rate for audio-visual clips meets the criterion for audio-visual integration. As can be seen in Equation 6, this indicator variable is *x*_*ind*_ > 1 if the hit rate for audio-visual clips exceeds the criterion for audio-visual integration and *x*_*ind*_ ≤ 1 if the hit rate for audio-visual clips can be explained by independent memory traces for the unimodal tracks of the clips.

6 $${x}_{ind}=\frac{\theta_{av}}{\theta_{crt}}$$

For the modeling itself, we used an informed prior on the indicator variable that prefers values around x_ind_ = 1 (i.e., neither preferring one of the different explanations; Equation 7) as well as mildly informed prior**s** for the recognition probabilities of auditory and visual tracks (i.e., to reflect the knowledge that performance in these conditions falls between chance level and ceiling; see Equation 8). Please note that *θ*_*crt*_ inherited its priors from *θ*_*a*_ and θ_v_, whereas *θ*_*av*_ inherited its prior from *θ*_*a*_, *θ*_*v*_, and *x*_*ind*_.

7 $${x}_{ind}\sim \mathrm{Normal}\left(\mu =1,\kern0.5em \sigma =.01\right)$$

8 $${\theta}_{a/v}\sim \mathrm{Beta}\left(\alpha =5.8,\kern0.5em \beta =4.2\right)$$

In order to calculate the joint probability given the data, we used the R package rjags (Plummer, 2016). We employed a Markov Chain Monte Carlo (MCMC) method (Gibbs Sampling; Gelfand & Smith, 1990) in order to derive samples from the posterior distribution. Following an adaption period of 1,000 samples as well as a burn-in period of 1,000 samples, four parallel chains performed 10,000 iterations with a thinning interval of 1. In order to check for convergence, autocorrelation, as well as effective sample size, we used the MCMC diagnostic tools included within the R package coda (Plummer et al., 2006).

### Results

**Fig. 3:**
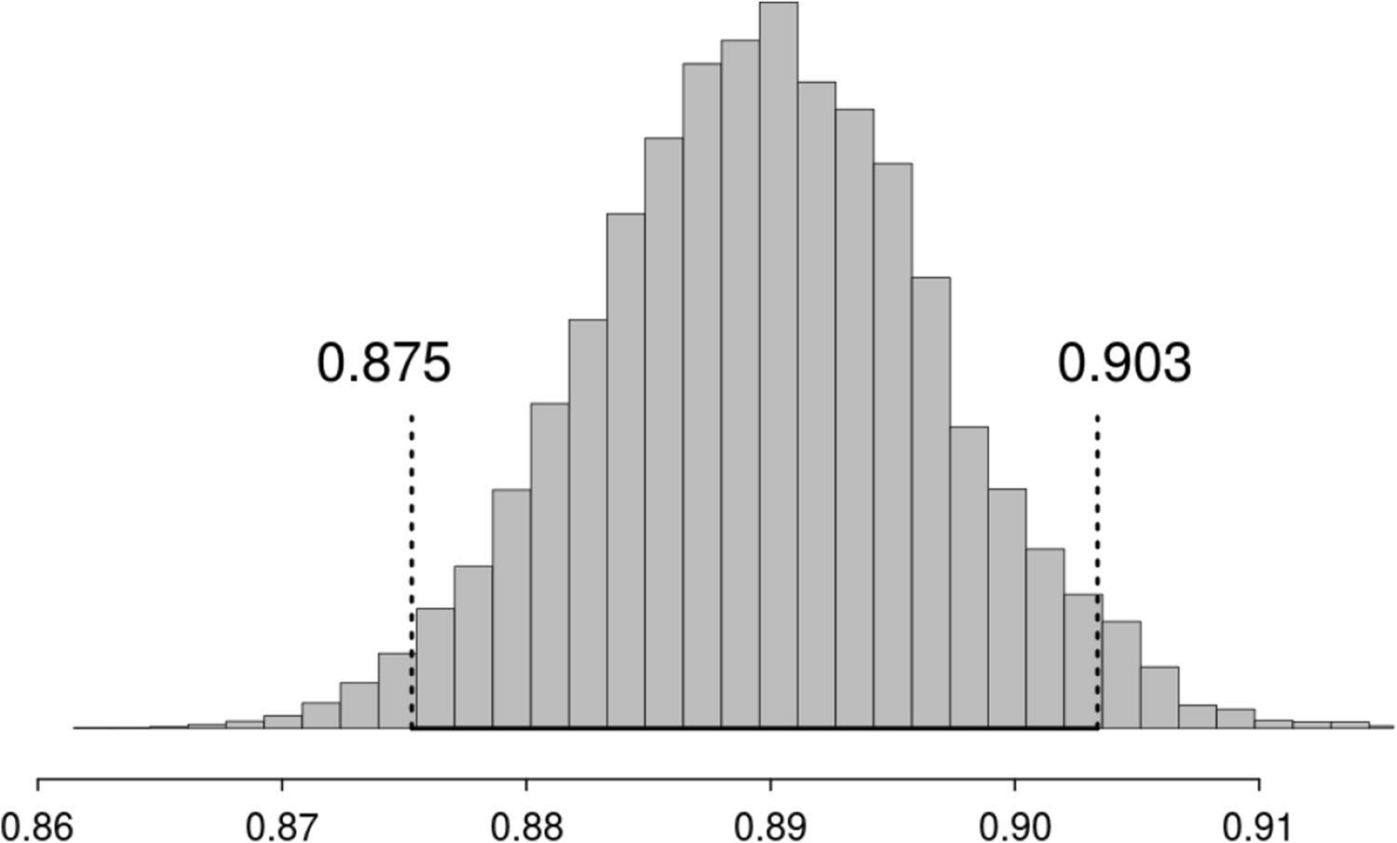
Highest density interval of the indicator variable for the simulated integration criterion. Values smaller than 1 indicate hit rates below the expected performance for independent storages for auditory and visual information. Values larger than 1 indicate that the hit rates exceed the expected performance based on independent storages for auditory and visual information. Note that the entire highest density interval ranges from 0.875 to 0.903

Our results were derived from the first MCMC chain. In order to determine whether differences in the observed means are meaningful, we calculated 95% highest density intervals (HDIs) for all reported parameters. As depicted in Fig. 3, our modeling approach indicated that the observed hit rates for audio-visual clips are clearly below the criterion for audio-visual integration proposed by Thompson and Paivio (1994). The parameter for the rate of correctly recognized audio-visual stimuli was *M* = 0.74, *SD* = 0.005, 95% HDI [0.73; 0.75], whereas the parameter for the estimation of the integration criterion was *M* = 0.83, *SD* = 0.003, 95% HDI [0.83; 0.84]. Consequently, the indicator variable x_ind_ revealed values smaller than 1, *M* = 0.89, *SD* = 0.007, 95% HDI [0.88; 0.90] (see Fig. 3).

### Discussion

The reanalysis of our previous data showed that the recognition performance for audio-visual scenes was substantially below the criterion of audio-visual integration proposed by Thompson and Paivio (1994). This finding is remarkable because most theories on audio-visual integration would expect performance above this threshold (signaling audio-visual integration) or around this threshold (signaling dual coding). Performance below the threshold (i.e., sub-additivity), however, requires further elaboration because this observation implies that our participants performed less accurately than predicted by independent memory traces for auditory and visual tracks.

So how can we explain this sub-additivity? In contrast to the predicted result patterns (performance at or above the threshold), performance below the threshold is less conclusive with regard to the question of whether auditory and visual information are integrated in long-term memory representations. On the one hand, research on the single-neuron level (Stein et al., 2009), as well as the level of neural activity in general (Sperdin et al., 2009) has suggested that sub-additivity might also reflect audio-visual integration as crossmodal depression (similar to crossmodal enhancement) indicates an interaction between the senses. On the other hand, however, this analogy does not necessarily hold true for memory processes as there are parsimonious alternative explanations that are independent of integration processes. For instance, with unimodal encoding, the total encoding time is twice as much as with audio-visual encoding, which might prevent audio-visual scenes from reaching the level of performance predicted by unimodal memory performance. Similarly, when auditory and visual track are stored separately, the necessity to do this at the same time for audio-visual clips would require allocating attentional resources between both modalities, which in return might result in less accurate memory performance than when both modalities are encoded in isolation.

In sum, this simulation does not provide strong evidence in favor of audio-visually integrated long-term memory representations. Due to the sub-additivity, however, a sub-optimal integration process remains possible, so that rejecting integration processes also might be premature. In the following experiments, we collect additional evidence, and we will return to the sub-additivity effect in the* General discussion*.

## Experiment 1a

Following the simulation study, which could not rule out or prove the existence of audio-visual integration processes with regard to long-term memory representations, we strived to provide further experimental evidence that could help to resolve this ambiguity. In the following experiments, we therefore tested a second prediction from audio-visually integrated memory performance for dynamic scenes by investigating effects of study-test congruency. This approach has been used intensively to study the nature of memory representations. For instance, it has been used to demonstrate that memory representations also include contextual information (e.g., Godden & Baddeley, 1975; Grant et al., 1998) as well as dynamic information of scenes (Buratto et al., 2009).

If auditory and visual information are integrated in long-term memory representations, a successful recognition of the scene should be more likely when the modality during the study session matches the modality of the testing session. In other words, a clip that has been studied audio-visually should be recognized more accurately when tested audio-visually rather than visually, and a clip that has been studied visually should be recognized more accurately when tested visually rather than audio-visually. As the first of these predictions could also be explained by an increase in retrieval cues (see below), the second of these predictions provides a strong test for the nature of the memory representation. If a scene has been studied purely visually, combining auditory and visual information during testing should result in mismatching representations, thus resulting in less accurate recognition performance than when the scene is tested only visually.

In contrast, if both auditory and visual information are not integrated in long-term memory representations of naturalistic scenes, memory accuracy should follow a function of the retrieval cues that could be used in the testing session. In this case, the condition with audio-visual clips during study and test should elicit the most accurate memory performance because the visual and the auditory track serve as independent retrieval cues in this condition. All other conditions should perform equally well because only the visual information is present during study and test in these conditions.

### Methods

#### Participants

Twenty-four students (all female; age 18–27 years) from the University of Tübingen participated in exchange for course credit or payment. The experimental procedure was approved by the institutional review board of the Leibniz-Institut für Wissensmedien, Tübingen, and all participants provided informed consent prior to their participation. This sample size was chosen to match previous experiments from our lab addressing audio-visual integration in long-term memory performance with similar materials (Meyerhoff & Huff, 2016). In these experiments, we observed large correlations between repeated measures that go along with rather large effect sizes (i.e., effects of the presentation modality ranged from η_p_^2^ = .40 to η_p_^2^ = .72). Transferred into power calculations, the sample size of 24 participants allows us to detect effects of η_p_^2^ = .28 reliably (α = .05, power (1- β) = .82).

#### Apparatus, stimuli, and procedure

The experiment was coded in Python using the PsychoPy libraries (Peirce, 2007). The stimuli were presented on a 23-in. LCD monitor (60 Hz, 1,920 × 1,080 pixels) controlled by a MacMini at an unrestricted viewing distance of approximately 60 cm.

The stimuli consisted of 1,200 brief clips from 50 Hollywood movies (1935–2008). From each of the movies, we extracted 24 clips, which were equally distributed across lengths of 3, 3.5, or 4 s (i.e., eight clips per length and movie). The clips were initially selected randomly from the movies with the only restriction that there were no filmic cuts within the clips (cinemetrix database; http://www.cinemetrix.lv/). Very few clips were replaced due to not carrying auditory and/or visual information, or the auditory information apparently mismatched the visual information (see Meyerhoff & Huff, 2016). Therefore, the final set of stimuli reflected a representative sample from various films and genres depicting a large variety of different visual scenes and auditory tracks that could easily be identified (including human speech, naturalistic sounds, and background music). Due to this selection procedure, it is likely that the clips differ among physical (e.g., luminance, visual activity) and psychological dimensions (e.g., salience, memorability). In order to eliminate any potentially confounding influence from the clips, we counterbalanced the assignment of each clip to the four experimental conditions as well as to the set of targets or foils across the subgroups of eight participants. This ensured that each clip was presented equally often in each condition and equally often as the target and foil. Further, there was a foil from the same movie (of the same length) for each target within the same experimental condition (in order to prevent participants from recognizing the movie rather than a particular clip).

The experiment was divided into a study and a test session separated by 24 h. The participants were instructed that they were participating in a memory experiment and that they would need to recognize the studied items in the testing session. During the study session (approx. 1 h), the participants attended to 600 of the clips (four clips of each length from each movie). 300 of the clips were presented with the matching sound track, whereas the other half were presented in silence. During the testing session (approx. 2 h), the participants saw the full set of stimuli and indicated after each one whether it had been presented during the study session by pressing the corresponding button on a keyboard (i.e., old/new recognition). Importantly, half of the stimuli that had been presented with the auditory track during the study session were presented without the auditory track during the test session, and half of the tracks that had been presented without the auditory track during the study session were accompanied by the auditory track during testing. Before the study session, the participants were informed that they would need to recognize the presented clips in either the same or the other modality condition (i.e., they were instructed to mark tracks as “old” even when they were aware of additional or lacking auditory information).

Taken together, our experiment therefore follows a 2 × 2 × 3 within-subject design, with modality being manipulated orthogonally between study (visual vs. audio-visual) and test (visual vs. audio-visual; i.e., a study-test congruency experiment). Further, we manipulated the clip length. The manipulation of the clip length was motivated by practical reasons. Whereas we aimed at testing clips without filmic cuts, it was difficult to find a sufficient number of clips lasting 4 s (the analysis will show that this had no effect on the outcome of the study). The clips were presented in their original resolution (768 × 576 pixels or 1,024 × 576 pixels) in the center of the screen.

Following the testing session, the participants received a list of the 50 movies and were asked to mark those they had seen within the last 5 years. Across all reported experiments, this number varied from none to 34 movies.^2^ Excluding familiar movies (for each participant individually) from the analysis did not affect any of the effects of the study or testing modality (nor any interactions). Therefore, we will not discuss this issue any further.

### Results

The results show that clips studied and tested audio-visually elicited the most accurate memory performance. However, there was no full study-test congruency effect as clips studied visually did not differ between audio-visual and visual testing.

**Fig. 4:**
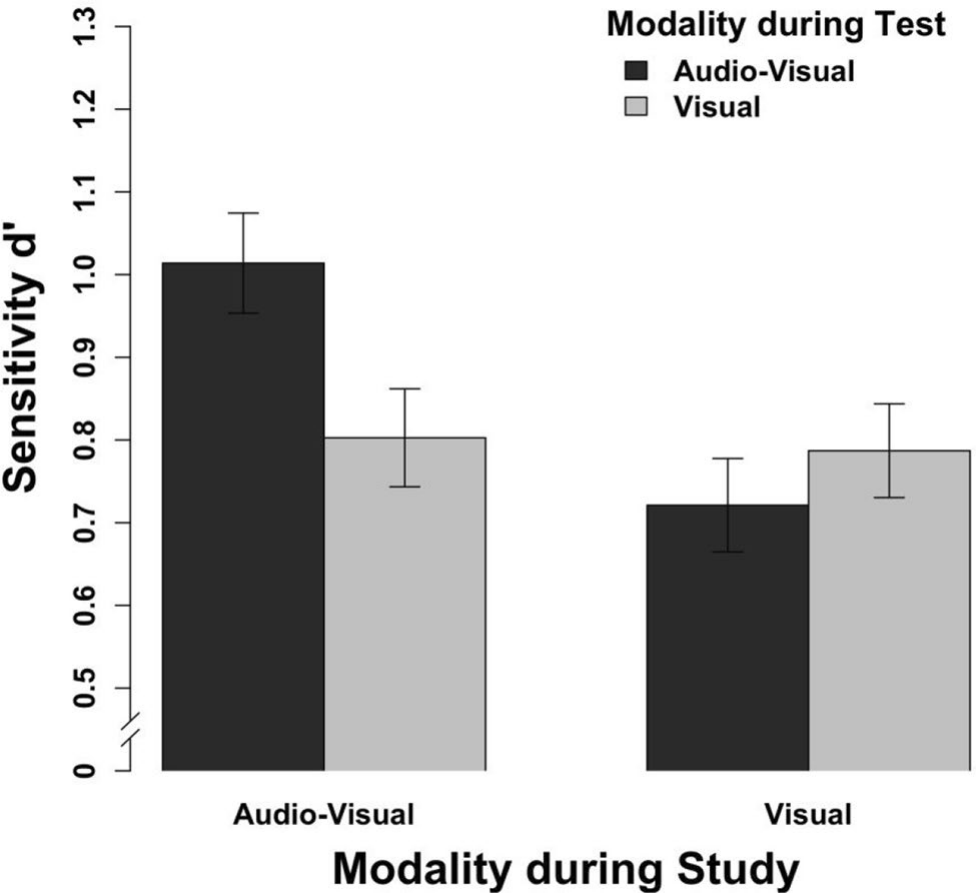
Results of Experiment 1a aggregated across the different clip lengths. The error bars indicate within-subject confidence intervals

We conducted a repeated-measures ANOVA with the study modality, the test modality, and clip lengths as the independent variables and the sensitivity measure d’ as the dependent variable (see Table 1 for means). Regarding the main effects, we observed more accurate memory performance with audio-visual than purely visual clips during the study session, *F*(1, 23) = 16.39, *p* < .001, η_p_^2^ = .42, 95% confidence interval (CI) [.11; .61], as well as increasing memory accuracy with an increasing length of the clips, *F*(2, 46) = 3.47, *p* = .040, η_p_^2^ = .13, 95% CI [0; .29]. However, as the CI includes 0, the increase from 3 to 4 s in clip duration might be too short to induce reliable effects. Further, the main effect modality during the testing session approached, but did not reach, significance, *F*(1, 23) = 3.77, *p* = .065. Most importantly, we observed an interaction between the study and the test modality (see Fig. 4), *F*(1, 23) = 10.18, *p* = .004, η_p_^2^ = .31, 95% CI [.04; .53]. However, this interaction did not indicate a full study-test congruency effect. Whereas clips that were studied audio-visually were recognized more accurately when the test was audio-visual rather than visual, *t*(23) = 3.57, *p* = .002, clips that were studied purely visually did not differ when they were tested audio-visually versus purely visually, *t*(23) = 1.21, *p* = .238. This pattern of results indicates that more retrieval cues present during study and test elicit a more accurate memory performance. Importantly, this does not require the assumption of an audio-visually integrated memory representation. None of the two- and three-way interactions including the clip length reached significance, all *Fs*(1, 23) < 2.49, all *ps* > .094.

**Table 1:** Results of all experiments (study/test) for different clip lengths

	Clip length					
	3 s		3.5 s		4 s	
	d'	c	d'	c	d'	c
	*M (SD)*	*M (SD)*	*M (SD)*	*M (SD)*	*M (SD)*	*M (SD)*
Experiment 1a						
av / av	0.88 (0.62)	0.31 (0.39)	1.05 (0.67)	0.27 (0.37)	1.17 (0.76)	0.25 (0.38)
av / v	0.83 (0.65)	0.49 (0.37)	0.82 (0.67)	0.40 (0.33)	0.80 (0.61)	0.37 (0.35)
v / av	0.70 (0.68)	0.49 (0.39)	0.70 (0.75)	0.36 (0.33)	0.79 (0.59)	0.38 (0.35)
v / v	0.78 (0.55)	0.46 (0.31)	0.74 (0.71)	0.41 (0.33)	0.86 (0.62)	0.43 (0.33)
Experiment 1b						
v / av	1.00 (0.38)	0.52 (0.50)	0.90 (0.36)	0.47 (0.45)	1.04 (0.40)	0.49 (0.47)
v / v	0.89 (0.34)	0.55 (0.45)	0.97 (0.41)	0.50 (0.46)	1.02 (0.43)	0.52 (0.44)
Experiment 2						
av / av	0.98 (0.82)	0.01 (0.30)	1.00 (0.95)	-0.07 (0.27)	1.05 (0.92)	-0.15 (0.36)
av / a	0.60 (0.54)	0.60 (0.24)	0.60 (0.60)	0.46 (0.31)	0.61 (0.73)	0.38 (0.34)
a / av	0.29 (0.41)	0.35 (0.27)	0.34 (0.55)	0.30 (0.40)	0.35 (0.54)	0.30 (0.36)
a / a	0.50 (0.51)	0.63 (0.35)	0.44 (0.61)	0.51 (0.25)	0.57 (0.70)	0.41 (0.24)
Experiment 3						
a / a	0.62 (0.49)	0.79 (0.43)	0.74 (0.48)	0.67 (0.39)	0.86 (0.51)	0.62 (0.45)
a / av	0.30 (0.35)	0.54 (0.36)	0.41 (0.49)	0.50 (0.40)	0.43 (0.42)	0.40 (0.38)
v / v	0.90 (0.47)	0.41 (0.41)	0.91 (0.55)	0.38 (0.47)	0.96 (0.56)	0.41 (0.45)
v / av	0.72 (0.44)	0.33 (0.42)	0.78 (0.51)	0.31 (0.40)	0.73 (0.48)	0.29 (0.47)
Experiment 4 (normal)						
av / av	1.32 (0.78)	0.12 (0.46)	1.33 (0.74)	-0.02 (0.44)	1.34 (0.71)	-0.03 (0.41)
av / v	0.97 (0.58)	0.08 (0.40)	1.12 (0.62)	0.07 (0.52)	1.11 (0.63)	0.15 (0.41)
v / av	0.83 (0.58)	0.21 (0.40)	0.97 (0.62)	0.12 (0.44)	1.01 (0.58)	0.15 (0.36)
v / v	0.97 (0.60)	0.18 (0.41)	1.18 (0.69)	0.11 (0.43)	1.18 (0.60)	0.17 (0.46)
Experiment 4 (degraded)						
av / av	0.87 (0.63)	0.35 (0.52)	0.90 (0.40)	0.30 (0.49)	1.00 (0.69)	0.25 (0.40)
av / v	0.42 (0.48)	0.54 (0.54)	0.47 (0.49)	0.54 (0.53)	0.42 (0.34)	0.42 (0.44)
v / av	0.35 (0.39)	0.60 (0.52)	0.40 (0.45)	0.52 (0.52)	0.37 (0.41)	0.43 (0.51)
v / v	0.50 (0.33)	0.56 (0.56)	0.35 (0.51)	0.49 (0.56)	0.49 (0.54)	0.55 (0.54)

*M* mean, *SD* standard deviation, *d'* sensitivity, *c* response criterion, *av* audio-visual, *v* visual, *a* auditory

The analyses of the response criterion c did not reveal anything of interest. In general, reduced sensitivity came along with a larger response bias, indicating that our participants tended to indicate a clip as new when they were not sure whether it had been presented in the study session. Therefore, we do not report these analyses in full detail; however, the corresponding values are summarized in Table 1.

## Experiment 1b

Experiment 1a did not reveal a study-test congruency effect because the testing modality was irrelevant for clips that had been studied purely visually. Although far from statistical significance, there was a numerical trend towards a full study-test congruency effect. In order to exclude the possibility that we did not observe the effect of the testing modality on clips that had been studied visually due to a lack of power, we replicated these two conditions with twice as many clips.

### Methods

#### Participants

Twenty-four new students (16 female; age 18–45 years) participated in Experiment 1b.

#### Apparatus, stimuli, and procedure

Apparatus, stimuli, and procedure were identical to Experiment 1a with the following exceptions. During the study session, 600 of the clips were presented visually. During the testing session, the participants indicated for all 1,200 clips whether they had been presented in the study session. One half of all clips in total as well as one half of the clips from the study session were presented audio-visually, whereas the remaining half of the clips were presented visually.

### Results

**Fig. 5:**
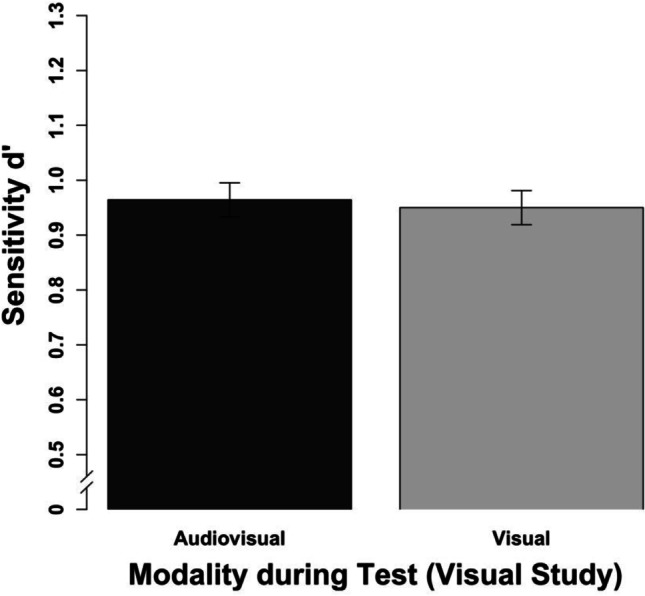
Results of Experiment 1b aggregated across the different clip lengths. The error bars indicate within-subject confidence intervals

In agreement with the results of Experiment 1a, we did not observe an impact of the testing modality for clips that had been studied visually (see Fig. 5). We conducted a repeated-measures ANOVA with the modality during the testing session as well as clip lengths as the independent variables and the sensitivity measure d’ as the dependent variable. Most importantly, there was no evidence that the modality during the testing session had any influence on memory performance when the clips were studied purely visually, *F*(1, 23) < 1, thus replicating the results from Experiment 1a. The main effect of the length of the clip approached but did not reach significance, *F*(2, 46) = 2.90, *p* = .065. Unexpectedly, the interaction between clip length and the modality during the testing session reached significance, *F*(2, 46) = 3.42, *p* = .041, η_p_^2^ = .13, 95% CI [0; .29]. A closer inspection of the mean values (see Table 1) showed that this appears to be unsystematic. Further, as the CI includes 0, this effect probably reflects noise, so we will not discuss it any further.

## Experiment 2

Experiments 1a and 1b revealed that visual and audio-visual testing elicited the same memory performance for clips that had been studied visually. There are at least two possible explanations for the absence of this effect. First, it is possible that information from different modalities is not integrated in long-term memory representations for naturalistic scenes but contribute to memory performance rather independently of each other. Alternatively, the absence of a study-test congruency effect might be a peculiarity of visual memory. From an ecological point of view, this seems reasonable as the auditory information might differ strikingly between study and test due to multiple reasons, such as the presence of additional noise or a varying distance to the scene. In order to differentiate between these two possibilities, we repeated Experiment 1a with auditory instead of visual scenes (i.e., auditory vs. audio-visual scenes). If the absence of study-test congruency effects is a peculiarity of visual memory, we should observe a full study-test congruency effect in this experiment. In contrast, the absence of a study-test congruency effect would signal that distinct modalities contribute to long-term memory performance rather independently of each other.

### Methods

#### Participants

Twenty-four new students (17 female; age 18–34 years) participated in Experiment 2.

#### Apparatus, stimuli, and procedure

Apparatus, stimuli, and procedure were identical to Experiment 1a with the following exceptions. Instead of visual scenes, we presented the auditory track of the same scenes in this experiment. During the study session, 300 of the clips were presented auditorily, whereas the remaining 300 clips were presented audio-visually. During the testing session, the assignment of the modality was reversed for one half of the clips. The 600 foils were divided equally between the two modality conditions.

### Results

Our results show a full study-test congruency effect for the combination of auditory and audio-visual clips. Without exception, a match in the modality conditions between study and test elicited more accurate performance than a corresponding mismatch.

**Fig. 6:**
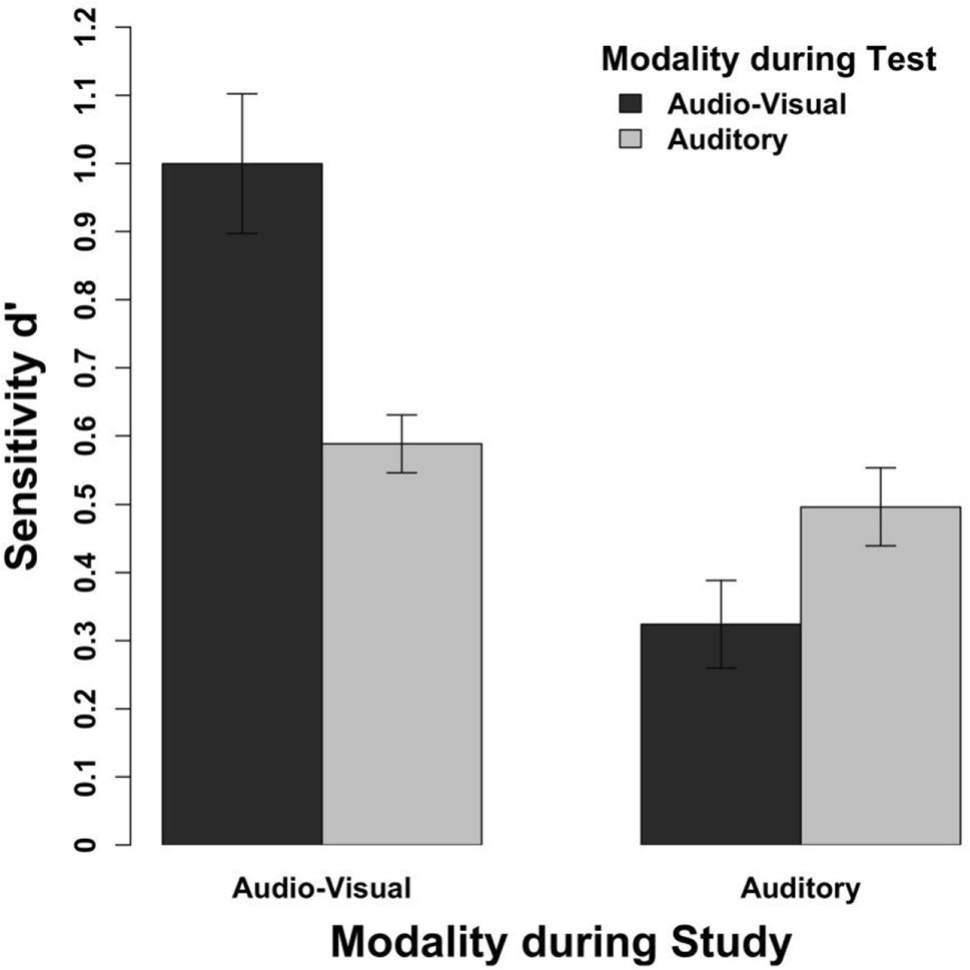
Results of Experiment 2 aggregated across the different clip lengths. The error bars indicate within-subject confidence intervals

We conducted a repeated-measures ANOVA with the study modality, the test modality, and clip lengths as the independent variables and the sensitivity measure d’ as the dependent variable (see Table 1). Regarding the main effects, we observed more accurate memory performance with audio-visual clips rather than auditory clips during the study session, *F*(1, 23) = 44.38, *p* < .001, η_p_^2^ = .66, 95% CI [.38; .78], as well as more accurate memory performance for audio-visual clips rather than auditory clips during the testing session, *F*(1, 23) = 11.67, *p* = .002, η_p_^2^ = .34, 95% CI [.05; .55]. Neither the main effect of clip length nor any two-way nor three-way interaction including length reached significance, all *F*s(2, 46) < 1. Most importantly, however, we observed an interaction between the study and the test modality (see Fig. 6), *F*(1, 23) = 32.87, *p* < .001, η_p_^2^ = .59, 95% CI [.28; .73]. In contrast to Experiment 1a, this interaction indicates a full study-test congruency effect because clips that were studied audio-visually elicited more accurate memory performance when they were tested audio-visually than auditorily, *t*(23) = 5.61, *p* < .001, and clips that were studied auditorily elicited more accurate memory performance when they were tested auditorily than audio-visually, *t*(23) = 3.95, *p* < .001. This finding indicates that study-test congruency effects for matching and mismatching modalities could be observed in general.

### Cross-experimental analysis

The presence of a full study-test congruency effect for auditory versus audio-visual scenes suggests that the consistent absence of the study-test congruency effect in Experiments 1a and 1b is a peculiarity of memory representations including visual information. However, because the presence versus absence of an effect between two experiments cannot be interpreted as an interaction (Nieuwenhuis, Forstmann, & Wagenmakers, 2011), we ran a cross-experimental analysis to prove whether the observed study-test congruency effects differ between Experiment 1a and Experiment 2a. Such an analysis between these experiments is possible as they are structurally equivalent. In both experiments, we presented half of the clips unimodally and the remaining half of the clips audio-visually. The only difference to a full between-subject design is the lack of a random assignment of the participants to the two experiments (however, both groups of participants stem from the same pool of students).

For the cross-experimental analysis, we ran a mixed ANOVA with experiment (between-subject; Exp. 1a vs. 2a), study modality (within-subject; unimodal vs. audio-visual) and the test modality (within-subject; unimodal vs. audio-visual) as independent variables and the sensitivity measure d’ as the dependent variable. Most importantly, we observed a significant three-way-interaction, *F*(1, 46) = 9.82, *p* = .003, η_p_^2^ = .18, 95% CI [.02; .36], indicating that the study-test congruency effects were differently pronounced between the two experiments. Furthermore, all two-way interactions as well as the main effects of study modality and test modality reached significance, all *Fs*(1, 46) > 18.23, all *ps* < .001, whereas the main effect of the experiment did not, *F*(1, 46) = 1.88, *p* = .177. Due to the three-way interaction, the results of this analysis are consistent with the interpretation that the recall of visual information is not susceptible to irrelevant additional auditory information during testing, but that auditory information is susceptible to irrelevant additional visual information during testing.

## Experiment 3

The results of Experiment 2 revealed that irrelevant additional visual information during testing is detrimental for an accurate recognition of auditory scenes. This pattern of results is relevant as it implies that recognition performance for auditory tracks might not operate independently from visual information as suggested by the dual-coding theory (i.e., even information that was not even present during encoding disrupts auditory recognition). In contrast, however, visual information could be recognized independently of irrelevant auditory information. Indeed, the cross-experimental analysis confirmed that this contrasts with the findings of Experiments 1a and 1b, which showed that irrelevant additional auditory information has little to no effect on the recognition performance for visual scenes.

Nevertheless, because cross-experimental analyses per definition do not include randomized sampling, we aimed to replicate the stronger susceptible influence of additional visual information on auditory recognition than vice versa while further probing the boundary conditions of this effect as well as the dual-coding framework. In the previous experiments, one modality was irrelevant for solving the task (i.e., in Experiments 1a and 1b visual information was sufficient whereas auditory information was irrelevant; in Experiment 2a, auditory information was sufficient whereas visual information was irrelevant). In contrast to this, we designed this experiment so that both modalities are relevant during the testing session. The participants studied auditory or visual clips in this experiment. During testing, half of the clips were presented in the same modality whereas the other half were presented audio-visually. For the unimodal clips, the participants simply reported whether the clip had been presented during the study session. For the audio-visual clips, the participants reported whether either the auditory or the visual track had been presented during the study session. Because the participants do not know whether the auditory or the visual track (if any) of an audio-visual clip had been presented during the study session, this implies that there are no irrelevant modalities in this experiment. Therefore, participants cannot simply focus on one modality during the test of audio-visual clips but need to process both of them simultaneously. Whereas a strong version of the dual-coding framework would predict that both tracks can be processed independently, the sub-additivity between the two modalities observed in the simulation study suggests a general decrease in recognition performance for auditory and visual tracks that are tested audio-visually. Most importantly, however, the detrimental effect of additional visual information on auditory recognition performance should be more pronounced than the detrimental effect (if any) of additional auditory information on visual recognition performance (i.e., a statistical interaction).

### Methods

#### Participants

Twenty-four new students (18 female; age 20–31 years) participated in Experiment 3.

#### Apparatus, stimuli, and procedure

Apparatus, stimuli, and procedure were identical to Experiment 1a with the following exceptions. During the study session, 300 of the clips were presented auditorily, and 300 clips were presented visually. During the testing session, half of these clips were presented in the same modality as during the study session, whereas the remaining half were presented together with the modality that was absent during the study session (i.e., audio-visually). The additional 600 foils were proportionally distributed across these modality conditions (i.e., 150 auditory foils, 150 visual foils, and 300 audio-visual foils).

### Results

Our results show that in general the recognition performance of unimodally studied tracks was lower when the additional irrelevant modality was present during the test. Most importantly, however, this susceptibility to the irrelevant additional modality is clearly more pronounced for auditory than for visual information.

**Fig. 7:**
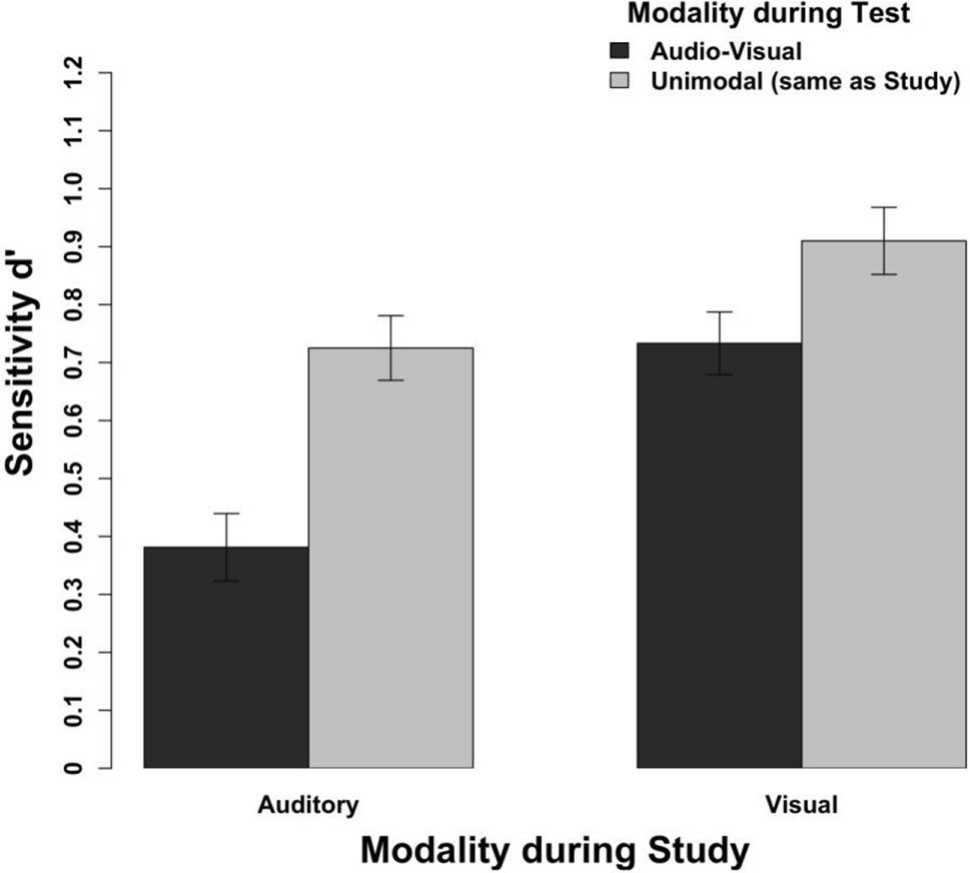
Results of Experiment 3 aggregated across the different clip lengths. The error bars indicate within-subject confidence intervals

We conducted a repeated-measures ANOVA with the study modality (auditory, visual), the presence of the irrelevant additional modality during the test (present, absent) and clip lengths as the independent variables, and the sensitivity measure d’ as the dependent variable (see Table 1). Regarding the main effects, we observed more accurate recognition performance for visually than auditorily studied clips, *F*(1, 23) = 36.95, *p* < .001, η_p_^2^ = .62, 95% CI [.32; .75], as well as more accurate recognition performance for unimodal testing (i.e., same modality as during study) than for audio-visual testing (i.e., with the irrelevant additional modality), *F*(1, 23) = 49.90, *p* < .001, η_p_^2^ = .68, 95% CI [.41; .80]. Neither the main effect of clip length nor any two-way nor three-way interaction including length reached significance, all *F*s(2,46) < 2.35, all *p*s > .107. Importantly, however, we observed an interaction between the modality during study and the presence of irrelevant additional modality during testing (see Fig. 7), *F*(1, 23) = 5.99, *p =* .022, η_p_^2^ = .21, 95% CI [.002; .45]. Although the effect size is rather small, this is consistent with the preceding cross-experimental analysis. This interaction shows that recognition performance for auditory clips is more susceptible for irrelevant additional visual information during testing than recognition performance for visual clips for irrelevant additional auditory information during testing. Nevertheless, the detrimental effect of the irrelevant additional modality during testing was present for auditorily, *t*(23) = 6.69, *p* < .001, as well as visually studied clips, *t*(23) = 3.55, *p* = .002. While this contradicts a completely independent processing of both modalities, it matches well with the results of the simulation study, which showed that recognition of audio-visual information is sub-additive relative to the isolated modalities.

## Experiment 4

The preceding experiments show that memory for visual scenes is less susceptible to additional auditory information during testing than memory for auditory scenes to additional visual information during testing. On the one hand, this suggests a dominant role of visual information for the formation of long-term memory representations of naturalistic scenes. On the other hand, however, it remains possible that the dominant role of visual information in our experiments arises from a higher discriminability or memorability of the visual tracks than the auditory tracks. In order to test this possibility, we repeated Experiment 1a with an additional manipulation of the quality of the visual tracks (normal vs. degraded). If the dominant role of visual information in our experiments arose from a superior discriminability and/or memorability of the visual track, visually degraded tracks should be more susceptible for additional auditory information during testing than regular visual tracks. In contrast, if visual information was generally dominant in memory representations, both degraded and normal tracks should be equally unsusceptible to additional auditory information during testing.

### Methods

#### Participants

Twenty-four new students (19 female; age 20–28 years) participated in Experiment 4.

#### Apparatus, stimuli, and procedure

Apparatus, stimuli, and procedure were identical to Experiment 1a with the following exceptions. Using the framework Frei0r (2018) with the Pixeliz0r filter set to a pixelization degree of .03 × .03, we generated visually degraded versions of our clips. As in Experiment 1a, 300 of the clips were presented visually whereas the remaining 300 clips were presented audio-visually during the study session (equally split between normal and visually degraded stimuli).^3^ During testing, half of the visually studied clips were presented audio-visually, and vice versa (equally split between normal and visually degraded stimuli). The additional 600 foils were proportionally distributed across all conditions.

### Results

**Fig. 8:**
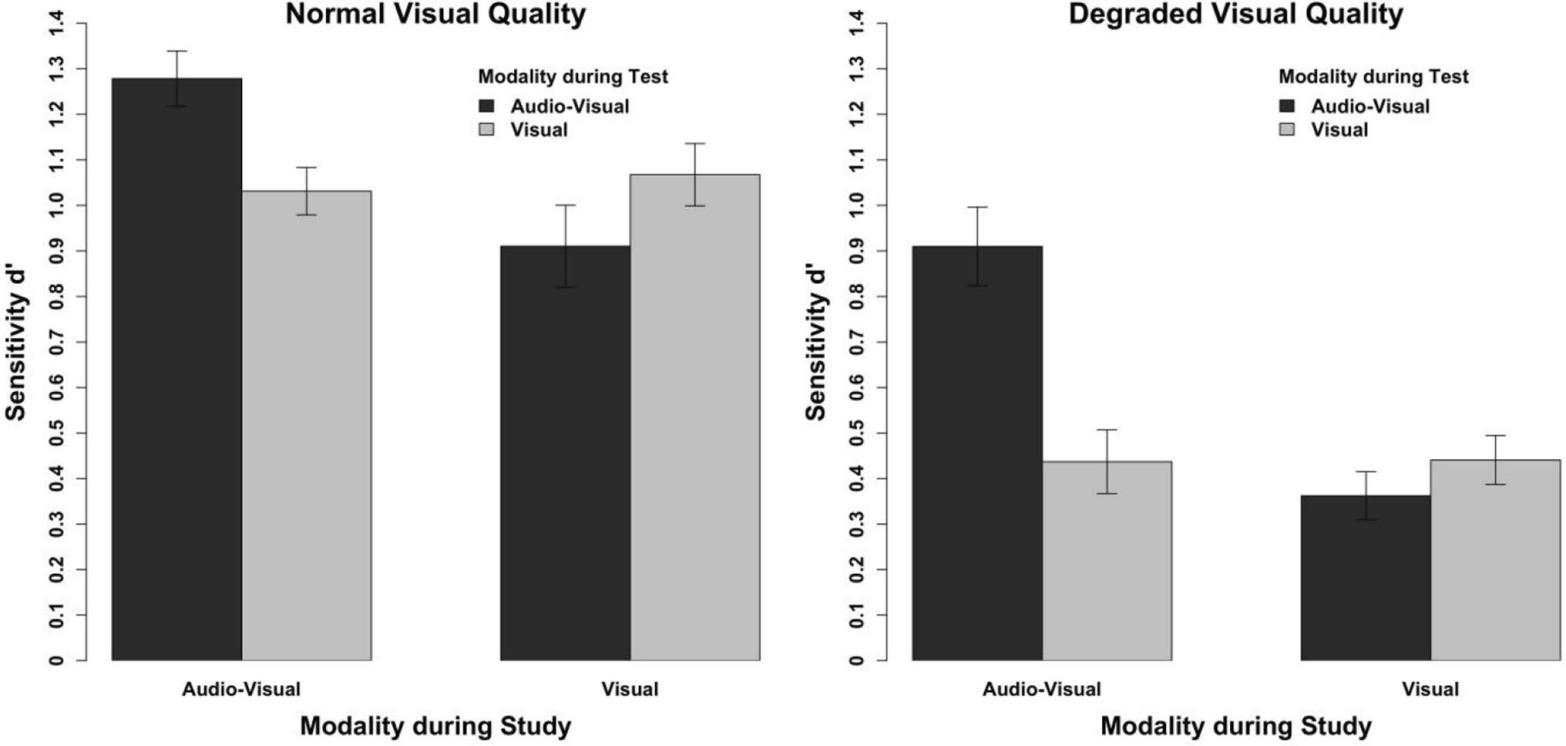
Results of Experiment 4 aggregated across the different clip lengths for clips of normal visual quality (left) and degraded visual quality (right). The error bars indicate within-subject confidence intervals

Our results confirm the conclusions from Experiments 1a, 1b, and 2. Most importantly, reducing the quality of the visual tracks did not increase the susceptibility to additional auditory information during testing (see Fig. 8).

We conducted a repeated-measures ANOVA with the visual quality (normal, degraded), the study modality (visual, audio-visual), the test modality (visual, audio-visual), and clip lengths as the independent variables and the sensitivity measure d’ as the dependent variable (see Table 1). Neither the four-way interaction nor any of the three-way interactions reached significance, all *F*s < 1.30, all *p*s > .283. Most importantly, we observed two two-way interactions (see Fig. 6). First, we observed an interaction between study modality and test modality, *F*(1, 23) = 60.98, *p* < .001, η_p_^2^ = .73, 95% CI [.48; .82]. This interaction indicates that a study-test congruency effect could be present in our data. Second, we observed an interaction between the test modality and the visual quality of the clips, *F*(1, 23) = 5.61, *p* = .026, η_p_^2^ = .20, 95% CI [0; .44]. This interaction indicates that the effect of the test modality differs between the normal and the degraded visual quality of the clips.

In a series of post hoc *t*-tests, we further pursued these interactions. Clips that were studied audio-visually elicited more accurate recognition performance when they were also tested audio-visually rather than visually in isolation for both the normal, *t*(23) = 5.97, *p* < .001, and the degraded visual quality, *t*(23) = 5.82, *p* < .001. In contrast, visually studied clips were not recognized more accurately when tested visually than audio-visually for both the normal, *t*(23) = 2.00, *p* = .058, and the degraded visual quality, *t*(23) = 1.80, *p* = .086. The overall pattern of these *t*-tests therefore suggests that the interaction between the study modality and the test modality arises from a partial but not a full study-test congruency effect (i.e., there is no detrimental effect of the addition of auditory information during testing). This replicates the findings of Experiments 1a and 1b. The interpretation of the interaction of the test modality and the visual quality is more complicated because the effect is so small that the lower bound of the CI of the effect size is 0. Further, there were no effects of the test modality in the post hoc tests for both visual qualities. If anything, however, the numerical effect is contrary to the prediction that an increased memorability induces the dominance of the visual information in memory representations (i.e., more pronounced at the normal rather than the degraded visual quality). Therefore, there is no evidence in the data that a reduction in the memorability of visual information alters the impact of additional auditory information on recognition accuracy.

None of the remaining two-way interactions reached significance, all *F*s < 2.11, all *p*s > .160. Regarding the main effects, we observed more accurate recognition performance for audio-visually than visually studied clips, *F*(1, 23) = 36.67, *p* < .001, η_p_^2^ = .61, 95% CI [.32; .75], as well as more accurate recognition performance for audio-visually than visually tested clips, *F*(1, 23) = 14.00, *p* = .001, η_p_^2^ = .38, 95% CI [.08; .59]. Finally, we observed a main effect of the visual quality, *F*(1, 23) = 129.85, *p* < .001, η_p_^2^ = .85, 95% CI [.69; .90], indicating that the pixelating procedure indeed reduced the memorability of the visual tracks. The main effect of the clip length did not reach significance, *F*(1, 23) = 2.36, *p* = .106.

## General discussion

The present simulation and experiments were set out to probe whether auditory and visual information of dynamic scenes are integrated in long-tern memory representations. With providing initial evidence for this question, we were aiming at distinguishing between various theoretical accounts that potentially could explain long-term memory performance for audio-visual scenes (see Fig. 1). With regard to this question, there were four key findings in the presented results that promote the understanding of the interplay of auditory and visual information in long-term memory. First, the simulation showed that recognition performance for audio-visual scenes is actually less accurate than one would expect based on the recognition performance for their unimodal counterparts. This finding shows that superior memory performance for audio-visual relative to unimodal scenes does not necessarily require the assumption of integrative processes. Second, we did not observe a full study-test congruency effect for audio-visual versus purely visual scenes. In particular, for scenes that had been studied visually, the testing modality was irrelevant (Experiments 1a, 1b, and 4). In this case, audio-visual integration predicts less accurate recognition performance for visually studied scenes that are tested audio-visually. Third, there is a full study-test congruency effect for auditory versus audio-visual scenes. This finding demonstrates not only that study-test congruency effects in principle could be observed in the context of audio-visual scenes (Experiment 2), but also suggests that visual memory differs from auditory memory in its susceptibility to the other modality (Experiment 3). Fourth, reducing the visual quality of the clips in order to reduce the memorability of the visual track did not increase the susceptibility of visual recognition performance to additional auditory information during testing (Experiment 4). We elaborate further on these key findings in the following two paragraphs. Then we discuss the impact of our findings for theorizing on the nature of long-term memory representations.

### The whole is smaller than the sum of its parts

Previous studies addressing the dual coding of auditory and visual information in the context of memory formation have observed that performance in the audio-visual condition reaches the accuracy predicted from independent memories for both modalities (Paivio & Csapo, 1973; Thompson & Paivio, 1994). However, in our analysis recognition performance was even lower than predicted by fully independent retrieval cues. There are two alternatives to how this effect could be reconciled within the framework of independent contributions of auditory and visual tracks to memory performance. First, presenting both modalities in isolation doubles the encoding duration. However, previous work from our lab (Meyerhoff & Huff, 2016) has shown that memory for audio-visual scenes is more accurate when the isolated visual and auditory tracks are presented immediately after each other rather than temporally separated. Because encoding durations are identical in both cases, this rules out an explanation solely based on encoding duration. Second, within the framework of independent retrieval cues, the simultaneous presentation of auditory and visual information might require splitting attention between the two modalities that could in return explain the lower recognition performance (e.g., Craik et al., 1996; Fernandes & Moscovitch, 2000; Kane & Engle, 2000).

The overall pattern of our results allows further speculation on this attentional splitting account. In Experiments 1a, 2, and 4, unimodal memory probes elicited roughly the same recognition performance whether they were studied audio-visually or unimodally. This indicates that there were no costs arising from attentional splitting during the encoding of audio-visual clips. In contrast, when both modalities are presented simultaneously during the testing session, processing both modalities appears to be associated with costs from attentional splitting. For audio-visual targets this becomes apparent from the sub-additive performance revealed by our simulation of audio-visual target recognition. For auditory studied clips, this becomes apparent in the lower recognition rates when the auditory target is embedded in additional visual information. The remarkable exception is visually studied targets embedded into additional auditory information during testing. For these targets, the additional auditory information does not impair recognition performance. This suggests that the costs from attentional splitting mostly draw upon auditory recognition (we discuss this in more detail within the next paragraph).

An asymmetric attentional splitting explanation also matches with one of our findings in Experiment 3. When the participants studied the scenes unimodally (i.e., auditory or visual), adding the remaining modality reduced recognition performance. Although this effect was larger for auditorily studied tracks, it was also present for visually studied tracks. Remarkably, such a detrimental effect of auditory information on visual recognition was present only in this experiment, not in Experiments 1a, 1b, and 4. A central difference between these experiments is that the additional auditory information was relevant in Experiment 3 (because it could also be an auditory target with additional visual information), but not in the other experiments. Thus, the necessity of attending to the visual and the auditory information (of the same clip) appears to lower recognition performance in general. This finding again matches remarkably well with the simulation study, which showed that recognition performance for audio-visual scenes is generally lower than predicted by the recognition probability of the individual modalities in isolation. Further, this finding is also in line with the assumption of a central attentional bottleneck (Tombu et al., 2011). Indeed, attentional involvement in one sensory stream such as detecting a target within a rapid series of images is capable of withdrawing attentional processing from the auditory stream and vice versa (Arnell & Jolicoeur, 1999; Ptito et al., 2008). Of course, our memory task did not require participants to detect targets, but it seems likely that salient events in one or the other sensory stream elicits similar effects.

It seems important to note that the overall recognition performance for audio-visual scenes does not rule out the possibility of an actual integration of both modalities. In fact, the detrimental effects of reduced encoding duration as well as effects of split attention might overshadow the potentially beneficial effects of audio-visual integration. Nevertheless, this analysis shows that the level of recognition performance for audio-visual scenes could be explained without the assumption of integrated auditory and visual information.

### Study-test congruency effects for audition, but not for vision

A further observation that is hard to reconcile with audio-visually integrated memory representations is the asymmetric study-test congruency for visual and auditory scenes relative to audio-visual scenes. Whereas memory representations of visual scenes were immune to the additional presence of auditory information during testing (when the auditory information could not be the target itself), the additional presence of visual information during testing interfered with memory for auditory scenes. In other words, visual information affected auditory memory but not vice versa. Importantly, this cannot be explained with a better memorability of the visual rather than the auditory tracks as a reduction in the visual quality of the tracks did not decrease the dominance of the visual information (Experiment 4). A comparable dominance of visual information has been reported in simple reaction time tasks in which the presence of visual stimuli undermined the perception of simultaneously presented auditory stimuli (i.e., Colavita effect; Colavita, 1974; Colavita & Weisberg, 1979; Egeth & Sager, 1977; Hecht, Reiner, & Karni, 2009). Importantly, the preference for visual information indeed reflects differences in the perceptual sensitivity rather than just a shift in the criterion towards the visual modality (Koppen et al., 2009), which is not restricted to simple displays but also emerges during object identification (Ngo et al., 2010; Yuval-Greenberg & Deouell, 2009) as well as in semantically meaningful stimuli (Sinnett et al., 2007; Stubblefield et al., 2013; but see also Koppen et al., 2008).

Importantly, a similar dominance of visual information has been reported for short memory durations (Posner, 1967; see also Posner et al., 1976). Particularly, non-visual information can be ignored (after corresponding instructions) more easily than visual information (Klein & Posner, 1974). Interestingly, previous work from the continuous recognition paradigm showed that semantically matching crossmodal information had a beneficial effect on subsequent unimodal recognition (Lehmann & Murray, 2005; Thelen et al., 2015; but see Pecher & Zeelenberg, 2022). Despite the obvious methodological differences between this paradigm and our current set of experiments, the most interesting factor is the temporal delay between study and test. In the continuous recognition paradigm, recognition occurred immediately and intermixed with initial encounters whereas there is a delay of a full day in our studies. For future research, this raises the interesting question whether auditory contributions to visual information might decay faster than the visual representation itself.

Of course, it remains undoubted that auditory information in principle is capable of altering visual processing (Sekuler et al., 1997; Shams et al., 2000). In order to explain under which circumstances one modality might dominate the other, Welch and Warren (1980) formulated the modality appropriateness principle. According to this principle, the task-specific acuity of the involved modalities affects how they are integrated (Bertelson et al., 2000; Vroomen et al., 2001). With regard to our results for long-term memory representations, such an interpretation would suggest that long-term memory for auditory information is so unreliable (see Cohen et al., 2009) that it has no effect on visual information in a weighted integration. In any case, what seems clear from the asymmetric occurrence of study-test congruency effects is that memory representations are not just the product of an equally weighted integration of auditory and visual information.

### Theoretical implications

As outlined in the *Introduction*, numerous attempts have been made to explain memory performance for audio-visual material. These explanations encompass an amodal integration of different sensory channels (Anderson, 1978; Kieras, 1978), redintegration (i.e., enhanced memory representations following audio-visual encoding that could be reactivated by unimodal retrieval cues; see Baddeley, 2007), dual coding (i.e., independent memory representations for auditory and visual components of the same stimuli; Thompson & Paivio, 1994), and triple coding (i.e., independent memories with additional associations between them; Glucksberg, 1984; Snodgrass, 1984). Interestingly, our results cannot be fully resolved by any of these theoretical accounts. First, the strong persistence of visual information is not in line with fully amodal memory representations. Second, audio-visually encoded scenes elicit the same memory performance in visual recognition as purely visually encoded scenes. In contrast, redintegration would have predicted more accurate memory performance for audio-visual scenes during visual recognition due to the supposedly more sophisticated memory representations. Finally, in contrast to the visual component, auditory information was not immune to the influence of the other modality, therefore questioning an independent memory trace for auditory information as proposed by the dual- and triple-coding models. As mentioned previously, we have studied dynamic scenes rather than static images mostly because we considered them to reflect a higher degree of ecological validity (while preserving experimental control). Given these striking differences in the material used, it might not be too surprising that previous accounts are not in full accord with our results.

Nevertheless, there are components of the dual-coding theory that fit in rather well with our results and should thus be incorporated into an explanation of memory for naturalistic scenes. First, as proposed by the dual-coding theory, auditory information enhanced memory performance, although there was no evidence of an actual integration of both of them. Second, visual information remained accessible rather independently even when auditory information was present during encoding (i.e., the absence of full study-test congruency effects for visual vs. audio-visual material). Considering the modality appropriateness principle (Welch & Warren, 1980) as well as the generally inferior auditory memory (Cohen et al., 2009), our study suggests that the core memory representation of dynamic scenes is visual in nature. What is more puzzling is the role of coinciding auditory information. Whereas auditory information is obviously stored (performance is well above chance level in all conditions), it appears not to be independent as soon as visual information is present. It thus seems more likely that auditory information – although not integrated with visual information – is associated with the visual information. We consider this to be a reformulation of the dual-coding theory with only one independent storage for visual information and an associated storage for additional auditory information.

## Conclusion

Studying naturalistic dynamic scenes, the present study shows that long-term recognition performance for audio-visual scenes does not require assumptions about audio-visually integrated memory representations. Instead, our study suggests that visual information is dominant in long-term memory for such naturalistic scenes and that additionally presented auditory information is associated with that visual information rather than being stored independently. These findings deviate from previous research that has been using simpler study material, thus calling for further investigation of long-term memory using as ecologically valid stimuli as possible.

### Open Practices Statement

All raw data and analysis scripts of the reported experiments are available at https://osf.io/hywcz/. None of the experiments was preregistered (data collection started in the year 2014).

## Data Availability

Open Access funding enabled and organized by Projekt DEAL. All data of the reported experiments are available at https://osf.io/hywcz/.

## Footnotes

1 Please note that we simulated hit rates whereas we analyzed sensitivity in our previous work (Meyerhoff & Huff, 2016). We decided so simulate only hit rates because there is a clear theoretically motivated criterion for audio-visual integration for hit rates but not for false alarm rates. Nevertheless, when we repeated the simulation with sensitivity as dependent variable based on the empirically observed false alarm rates in the audio-visual condition, the results and conclusions were identical to the simulation of the hit rates (the full simulation script is available at https://osf.io/hywcz/).

2 Familiarity data are missing for one participant.

3 Due to a codec error, we lost data for one clip for eight participants. Due to the counterbalancing procedure this affects all modality conditions equally.

## Code availability

All analysis scripts of the reported experiments are available at https://osf.io/hywcz/.
